# Microfluidic Assays for CD4 T Lymphocyte Counting: A Review

**DOI:** 10.3390/bios15010033

**Published:** 2025-01-09

**Authors:** Zhuolun Meng, Hassan Raji, Mahtab Kokabi, Deng Zou, James Chan, Qihao Liu, Ruifeng Zhang, Mehdi Javanmard

**Affiliations:** Electrical and Computer Engineering, Rutgers University-New Brunswick, 94 Brett Road, Piscataway, NJ 08854, USA; zhuolun.meng@rutgers.edu (Z.M.); hr322@soe.rutgers.edu (H.R.); mk1903@scarletmail.rutgers.edu (M.K.);

**Keywords:** ASSURED criteria, CD4 counting, HIV/AIDS, microfluidics, point-of-care

## Abstract

CD4 T lymphocytes play a key role in initiating the adaptive immune response, releasing cytokines that mediate numerous signal transduction pathways across the immune system. Therefore, CD4 T cell counts are widely used as an indicator of overall immunological health. HIV, one of the leading causes of death in the developing world, specifically targets and gradually depletes CD4 cells, making CD4 counts a critical metric for monitoring disease progression. As a result, accurately counting CD4 cells represents a pressing challenge in global healthcare. Flow cytometry remains the gold standard for enumerating CD4 T cells; however, flow cytometers are expensive, difficult to transport, and require skilled medical staff to prepare samples, operate the equipment, and interpret results. This highlights the critical need for novel, rapid, cost-effective, and portable methods of CD4 enumeration that are suitable for deployment in resource-limited countries. This review will survey and analyze emerging research in CD4 counting, with a focus on microfluidic systems, which represent a promising area of investigation.

## 1. Introduction

HIV/AIDS remains a critical global health issue, claiming approximately 40.1 million lives worldwide. In 2021 alone, 1.5 million new cases and 650,000 deaths were reported [1], with an estimated 38.4 million individuals currently living with HIV, two-thirds (25.6 million) residing in the WHO African Region [1]. CD4 T lymphocytes play a critical role in orchestrating immune responses, and their depletion is a hallmark of HIV infection [2,3]. Measuring CD4 counts provides crucial information for assessing disease progression, initiating antiretroviral therapy (ART), and monitoring immune recovery. Current WHO guidelines recommend CD4 testing at baseline for all newly diagnosed HIV patients and periodically for those with advanced disease or suspected treatment failure. While viral load testing is increasingly prioritized, CD4 testing remains indispensable in resource-limited settings where viral load testing may not be readily available [4].

CD4 metrics—absolute count, CD4%, and the CD4/CD8 ratio—are essential for assessing immune health and managing HIV/AIDS. Each metric serves a unique role in HIV care. For instance, CD4% indicates the proportion of CD4 cells among total lymphocytes and is particularly useful in pediatric cases where absolute counts vary with age. The CD4/CD8 ratio has emerged as a marker for immune activation and aging, with relevance for non-AIDS comorbidities in treated HIV patients [5]. This review focuses on the absolute CD4 count, the most commonly used metric, which measures the concentration of CD4 cells in the blood, which values below 200 cells/mm^3^ indicate AIDS [1,6,7]. CD4 testing is not routinely required for the general public but is critical for HIV-positive individuals. Baseline testing determines the stage of HIV and guides ART initiation [8]. Follow-up testing is essential for patients with advanced HIV (CD4 ≤200 cells/mm^3^) or opportunistic infections to monitor immune reconstitution and guide prophylaxis. For stable patients with suppressed viral loads, the WHO suggests that routine CD4 monitoring may not be necessary, especially if viral load testing is available [9]. The precision required for CD4 testing depends on clinical goals. Thresholds such as CD4 ≤200 cells/mm^3^ (indicating AIDS) are critical for decision-making, while thresholds of ≤350 cells/mm^3^ reflect evidence supporting earlier intervention [10]. Most recently, ≤500 cells/mm^3^ has been adopted as studies suggest benefits from earlier treatment initiation [10]. Semi-quantitative approaches (e.g., below or above thresholds) may suffice in some contexts, provided they correlate reliably with flow cytometry [11]. However, accurate absolute counts remain crucial for baseline assessments and monitoring immune recovery [10].

Flow cytometry, the gold standard for CD4 enumeration, uses immunolabeling techniques to achieve high accuracy and reliability [12,13,14,15,16]. However, its cost, operational complexity, and dependence on skilled medical staff limit accessibility in resource-limited settings [13,17,18,19]. Portable flow cytometers have improved accessibility, but devices like the BD FACSCount™ remain impractical for widespread POC use due to their size and medical requirements [13].

In response to these limitations, microfluidic technologies have emerged as transformative solutions for CD4 enumeration. These systems align with the ASSURED criteria (Affordable, Sensitive, Specific, User-friendly, Rapid and Robust, Equipment-free, and Deliverable) for medical diagnostics [20,21,22,23,24]. Innovations such as soft microfluidic channels [25,26,27] and disposable microfluidic devices [28,29,30,31,32,33] have enhanced practicality by reducing contamination risks and enabling easy fabrication.

Microfluidic technology has significantly advanced the development of point-of-care (POC) assays, providing practical alternatives to traditional flow cytometry. The choice between POC and centralized testing depends on local healthcare infrastructure and patient accessibility. While centralized testing offers higher throughput and precision, it requires sample transportation, which can cause delays and risks of sample degradation [34]. CD4 counts are susceptible to time- and temperature-related decay, particularly in whole blood samples, necessitating immediate processing or using stabilized transport media. POC tests, by contrast, provide immediate, on-site results, reducing turnaround times and enhancing patient care in remote regions [35].

These POC devices reduce costs, save time, and empower patients to monitor their health, promoting better understanding of disease progression and enabling timely decisions for HIV/AIDS management. This review examines current academic and commercial CD4 diagnostic methods, focusing on microfluidic devices and POC solutions. CD4 cell sensing methods are categorized as electrical or optical. Electrical sensing measures impedance changes as cells move through an aperture or bind to a target area, while optical sensing employs fluorescence- or non-fluorescence-based imaging to count CD4 cells. This review highlights these approaches and discusses innovative and unconventional sensing techniques.

## 2. Electrical Impedance Sensing

Electrical impedance sensing measures the opposition to an electrical current as it passes through a microfluidic channel containing cells. This section reviews three key approaches: Coulter principle-based methods, label-free impedance sensing, and electrical impedance spectroscopy (EIS). These techniques provide diverse CD4 T cell enumeration solutions and highlight innovations aimed at resource-limited settings.

### 2.1. Coulter Principle-Based Impedance Sensing

**Figure 1:**
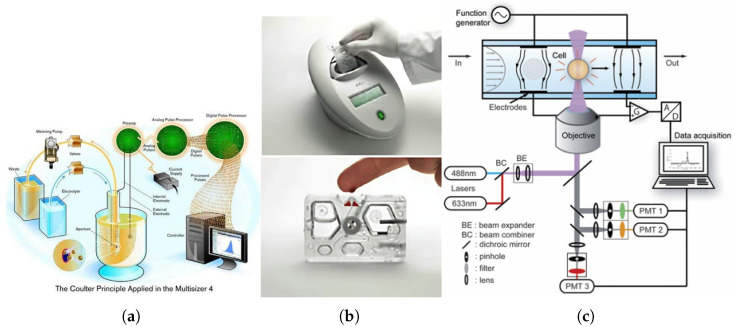
(**a**) Coulter principle-based sensing technique invented by Wallace H. Coulter [45]. (**b**) Top: Chempaq XBC blood counter. Bottom: Disposable cassette for blood collection [46]. (**c**) Improved Coulter Principle-based microfluidic device applied with multiple frequencies [37].

Coulter principle-based sensing relies on detecting changes in electrical impedance as cells pass through a small aperture. As the first technique to enable rapid analysis of single-cell electrical properties [36,37], it paved the way for high-throughput cell counting. However, it faces significant limitations for CD4+ T cell counting. Specifically, Coulter-based systems cannot inherently differentiate lymphocyte subtypes, such as CD4+ and CD8+ T cells, as they measure cell size and general electrical properties rather than specific surface markers essential for subtype identification [38,39,40]. Additional challenges, including aperture clogging, sensitivity to similarly sized particles, and extensive sample preparation requirements, further hinder its applicability to CD4 counting [40,41]. Recent advancements, such as multi-frequency measurements, have improved subtype identification and reduced sample preparation needs, increasing the technique’s suitability for point-of-care applications. As shown in Figure 1a, this technique measures changes in electrical impedance as particles pass through a small aperture, with impedance momentarily increasing for each particle [36]. Despite these improvements, the inability to identify specific surface markers remains a limitation for CD4+ T cell counting. For example, devices like the Chempaq XBC (eXpress Blood Counter, Chempaq A/S, Hirsemarken 1B, 3520 Farum, Denmark), shown in Figure 1b, provide total WBC counts with a three-part differential (Neutrophils, Lymphocytes, and Monocytes) but cannot reliably differentiate CD4+ T cells without additional markers [42,43,44]. Rao et al. evaluated the Chempaq XBC for hemoglobin and leukocyte counting, reporting accurate hematologic data suitable for high-throughput applications [41]. Furthermore, as illustrated in Figure 1c, Holmes et al. demonstrated that applying multi-frequency measurements could provide additional information about cell membrane properties and internal structure, enhancing subtypes differentiation [37,39]. By using antibody-coated beads to bind to CD4+ T cells, the technique changes the electrical properties of labeled cells, improving their differentiation from other cell types in mixed populations [37,39]. Combined with red blood cell lysis techniques, these advancements reduce preparation requirements and streamline the process for point-of-care applications [37,39,41]. Coulter-based impedance sensing provides fast, high-throughput analysis but remains limited for subtype-specific applications without additional markers.

### 2.2. Label-Free Impedance Sensing

Label-free impedance sensing simplifies CD4 enumeration by measuring changes in electrical resistance caused by cell size and membrane properties without requiring fluorescent labels or antibodies. Recent innovations include the use of fluidic electrodes made from KCl solutions, which eliminate the need for traditional metal electrodes and reduce costs while maintaining high sensitivity (e.g., detecting as few as 10 cells/µL). These approaches align with ASSURED criteria, making them ideal for resource-limited settings [47,48].

Wang et al. introduced a microfluidic device utilizing hydrodynamic focusing, where two sheath flows of KCl solution act as fluidic electrodes to guide the cell suspension through the channel [47,48]. The device applies a low-voltage (DC < 2V) electric field and measures impedance changes caused by ions and biomolecules released from lysed CD4+ T cells. The system demonstrated a linear relationship (R² = 0.97) between the logarithmic value of cell concentration and impedance, achieving a detection limit of 10 cells/µL. CD4+ cells were separated from whole blood samples prior to analysis to ensure specificity and accuracy [47,48].

**Figure 2:**
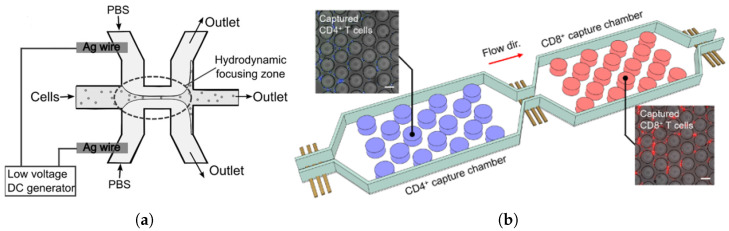
(**a**) Schematic representation of microfluidic structure and hydrodynamic focusing zone [47]. (**b**) A schematic of the device showing the layout of capture chambers designed to capture CD4+ and CD8+ T cells and the sensors monitoring cell capture. Insets show fluorescently labeled CD4+ (left) and CD8+ (right) cells after capture. Scale bar, 50 μm [49].

Arifuzzman et al. further demonstrated an autonomous microchip capable of analyzing immune cell subtypes without conventional labeling methods during cell permeabilization, facilitating electronic quantification of immunophenotypic characteristics [49]. This device uses microfluidic chambers functionalized with surface markers specific to capture target cells. As shown in Figure 2b, electrical impedance sensing measures resistance changes as cells pass through microchannels. Real-time data are fed into an algorithm to calculate cell subpopulation fractions based on immunocapture statistics [49]. While promising for point-of-care diagnostics in resource-limited settings, challenges remain in reducing hydraulic resistance and enabling faster processing. Additionally, integrating multi-frequency impedance analysis could help distinguish cell subtypes with similar electrical properties, making it adaptable for diverse cell populations. However, this device’s specific surface marker preparation limits its versatility for broader immune profiling applications [49]. Label-free impedance is cost-effective, autonomous, and well suited for portable diagnostics in decentralized healthcare environments.

### 2.3. Electrical Impedance Spectroscopy (EIS) Sensing

**Figure 3:**
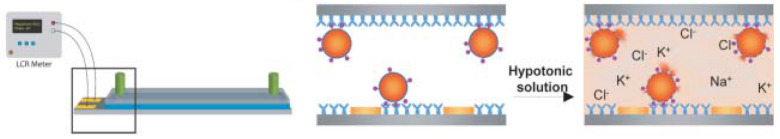
Left: Schematic of the impedance measurement setup. Samples are delivered into the microchannels through an inlet (green) using a syringe pump, with impedance measured by an LCR meter. Right: Illustration of cell ion release measured using impedance spectroscopy. Target cells isolated within a microfluidic device are lysed to release intracellular ions, increasing bulk conductance, and monitored with surface-patterned electrodes and impedance spectroscopy to quantify cell numbers [50].

EIS extends traditional impedance sensing by analyzing frequency-dependent electrical properties of cells [50]. This technique is particularly suited for highly sensitive CD4 detection in low-conductivity environments. However, challenges remain in minimizing interference from complex biological samples. Cheng et al. developed a microfluidic device that immobilizes CD4+ cells on patterned surface electrodes using anti-CD4 antibodies, as shown in Figure 3 [50]. Unbound cells and contaminants are removed via PBS washes containing BSA and EDTA to enhance specificity. This electrical method counts cells by measuring changes in the conductivity of the surrounding medium triggered by ions released from lysed, surface-immobilized cells within a microfluidic channel. Immobilized cells are lysed using a low-conductivity hypotonic solution, and the resulting changes in impedance are measured with surface-patterned electrodes to detect and quantify cell numbers. The conductance of the solution increases linearly with the number of lysed cells, achieving a detection limit of 20 cells/µL. (The equivalence of 200 cells/mm^3^ to approximately 20 cells/µL^3^ assumes a standard 1:10 dilution of blood, which is commonly used in certain CD4 testing methodologies.) This approach simplifies cell quantification and provides a reliable method for CD4 enumeration in microfluidic devices. However, EIS systems require low-conductivity environments, which may be affected by biological sample complexity [51]. Despite this limitation, EIS remains highly sensitive and specific, making it an ideal choice for controlled diagnostic environments.

Table 1 summarizes the strengths and limitations of these electrical impedance sensing techniques. Coulter principle-based impedance sensing offers fast, high-throughput analysis but struggles with subtype differentiation. Label-free impedance sensing provides affordable, autonomous solutions suitable for resource-limited settings, while EIS achieves high sensitivity but requires controlled environments. Collectively, these methods demonstrate significant potential for advancing CD4 counting technologies, particularly in low-resource settings.

## 3. Optical Sensing

Optical sensing methods utilize light-based detection techniques to quantify CD4 T lymphocytes in microfluidic assays. These approaches offer high sensitivity and specificity, making them well suited for point-of-care (POC) diagnostics. This section reviews fluorescence-based, imaging-based, absorbance-based, and colorimetric sensing techniques, highlighting their applications, limitations, and advancements in resource-limited settings.

### 3.1. Fluorescence-Based Optical Sensing

Fluorescence-based methods employ fluorophore-conjugated antibodies to label CD4 cells, enabling highly specific detection [52]. Upon excitation by a light source, typically a laser, the fluorophores emit distinct fluorescent signals captured and quantified, allowing for high sensitivity even in samples with low cell counts [53]. Most fluorescence-based sensing approaches require fluorescence imaging techniques. For example, devices such as the BD FACSPresto integrate fluorescence imaging and absorbance readings, providing accurate CD4 counts for POC diagnostics [54]. Other systems, like the PIMA CD4 analyzer, forego imaging by using photodetectors to measure fluorescence intensity [11]. While these techniques excel in sensitivity, they often require precise optical alignment and can suffer from signal interference in complex biological samples.

**Figure 4:**
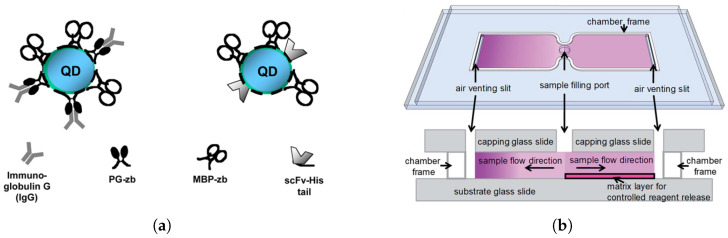

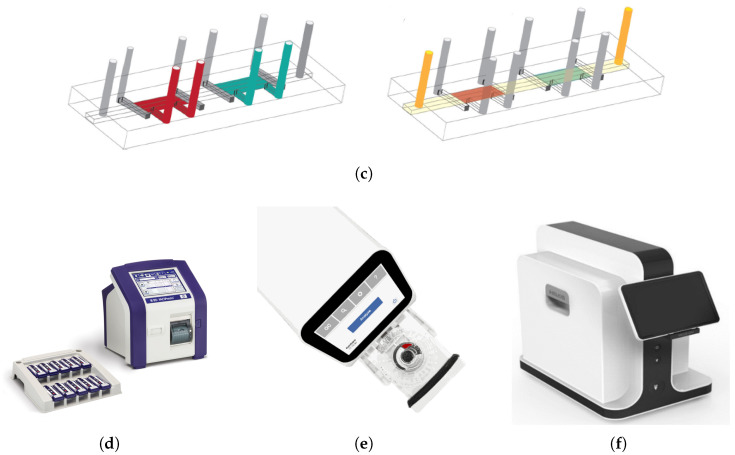
(**a**) Quantum dots (QDs) with antibodies for CD4 detection, offering compact and targeted binding [57]. (**b**) Perspective view of a simple glass chip with two chambers for capillary flow [58]. (**c**) Tandem affinity microfluidic system for CD4/CD8 ratio quantification, comparable to conventional flow cytometry [59]. (**d**) BD FACSPresto device [60]. (**e**) PointCare NOW™ system [61]. (**f**) Helios CD4 Analyzer with a helical channel design [62].

Quantum dots (QDs), as shown in Figure 4a, provide enhanced stability, brightness, and multiplexing capabilities due to their broad excitation and narrow emission spectra [55,56]. These properties enable the simultaneous detection of multiple cellular markers with minimal spectral overlap, significantly improving the quantification of CD4+ T cells. For instance, a portable microfluidic leveraging QDs achieved a high correlation (R² = 0.97) with conventional flow cytometry for counting CD4+ lymphocytes from whole blood [56,57]. Despite their potential, QDs remain underutilized for quantitative CD4 counting in disposable devices, a promising area for further development.

Innovative approaches like inkjet-printed polysaccharide matrices have also emerged. Shown in Figure 4b, this method incorporates fluorescent antibodies within a microfluidic CD4 counting chamber, enabling precise cell labeling and quantification with a cost-effective, long-lasting design [58]. This matrix, composed of gellan and trehalose, supports controlled antibody release, sustaining functionality in fluorescent assays for up to three months [58]. When a blood sample flows through, the matrix releases antibody conjugates, allowing precise CD4 labeling as the sample progresses through the device via capillary action. This configuration presents a cost-effective and efficient solution for POC CD4 counting [58]. In Figure 4c, another method involves a tandem affinity microfluidic system for CD4/CD8 ratio measurement, allowing simultaneous capture and tagging of both CD4 and CD8 cells, with results showing strong correlation with flow cytometry (R² = 0.97), underscoring its suitability as an affordable diagnostic tool in low-resource settings [59].

As shown in Table 2, several commercially available devices leverage fluorescence-based technology for CD4 T cell counting and have been widely applied in point-of-care settings, showing similar efficacy and accuracy to lab-based flow cytometry [63,64,65]. The BD FACSPresto (Becton, Dickinson and Company, PLC, 1 Becton Drive, Franklin Lakes, NJ 07417, USA), shown in Figure 4d and authorized by the World Health Organization in 2014 [60,66], is designed for low-resource settings; this system incorporates a single-use disposable cartridge, enabling rapid, on-site testing [44]. The BD FACSPresto requires a small volume of whole blood (50 µL), which is introduced into a single-use disposable cartridge preloaded with reagents, including fluorophore-conjugated antibodies specific to CD4 molecules on T cells. Once the cartridge is inserted into the analyzer, the fluorophore-conjugated antibodies bind selectively to CD4 molecules on the surface of T cells. Inside the analyzer, the sample is exposed to a light source that excites the fluorophores, causing them to emit light at specific wavelengths. The emitted fluorescence is captured and quantified by an imaging system, which calculates the number of CD4+ T cells in the sample with high specificity and accuracy. Studies indicate robust performance across various clinical settings, although sensitivity may be slightly lower than laboratory-based flow cytometry, potentially missing up to 20% of patients needing treatment in field settings [67,68]. The BD FACSPresto system has been successfully deployed in sub-Saharan Africa, where it demonstrated robust performance in HIV monitoring despite field limitations. Studies revealed up to 80% sensitivity in low-resource settings, though technical challenges like cartridge disposal logistics remain [69].

The PointCare NOW™ (PointCare Technologies, Inc, 257 Simarano Drive, Marlborough, MA 01752, USA), shown in Figure 4e and introduced in 2012, utilizes fluorescence-based detection similar to the BD FACSPresto. It requires 50 µL of whole blood mixed with fluorophore-conjugated antibodies that specifically bind to CD4 molecules on T cells [64]. However, PointCare NOW™ enhances functionality by integrating fluorescence detection with impedance sensing, enabling it to provide comprehensive blood count analysis. Impedance sensing measures the size and count of white blood cells, offering additional hematological parameters alongside CD4 counts. While the device is marketed for HIV/AIDS diagnostics, studies have reported a consistent positive bias in CD4 counts, raising concerns about its precision for clinical management. Independent evaluations suggest that it may not be suitable for all clinical settings [63].

An emerging fluorescence-based approach, “Nut and Bolt Microfluidics”, utilizes a helical minichannel within a cylindrical sample cartridge [62]. The Helios CD4 Analyzer, shown in Figure 4f, integrates fluorescence-based detection with a disposable helical cartridge designed for 20–50 µL of whole blood. Similar to the BD FACSPresto and PointCare NOW™, the Helios (Standard BioTools Inc. 2 Tower Place, Suite 2000, South San Francisco, CA 94080, USA) uses fluorophore-conjugated antibodies to label CD4+ T cells specifically. However, it distinguishes itself by employing a CCD camera to detect emitted fluorescence signals, offering precise quantification of labeled cells. The cylindrical cartridge features a spiraling channel, which ensures uniform mixing of the blood sample and reagents, minimizing reagent consumption and improving consistency. The system is powered by a single rotating motor, simplifying the electromechanical design and enhancing reliability in point-of-care (POC) settings, particularly in resource-limited environments. This innovative design streamlines operation while maintaining accuracy, positioning the Helios as a promising tool for accessible and efficient CD4+ T cell enumeration.

Additionally, comparative studies demonstrate promising agreement with the widely used PIMA CD4 analyzer (Abbott Rapid Diagnostics Jena GmbH, Orlaweg 1, D-07743 Jena, Germany), which shares the same basic fluorescence-based detection principle and suggests its potential as a low-cost, high-throughput diagnostic device [62]. The PIMA CD4 analyzer was assessed in South Africa using capillary blood sampling, which provides immediate CD4 counts, facilitating timely antiretroviral therapy initiation in resource-limited settings [11].

### 3.2. Imaging-Based Optical Sensing

Imaging-based techniques utilize high-resolution cameras or sensors to analyze cell morphology, offering significant potential for POC applications. These methods simplify optical setups while maintaining accuracy through innovations like machine learning-assisted cytometry.

**Figure 5:**
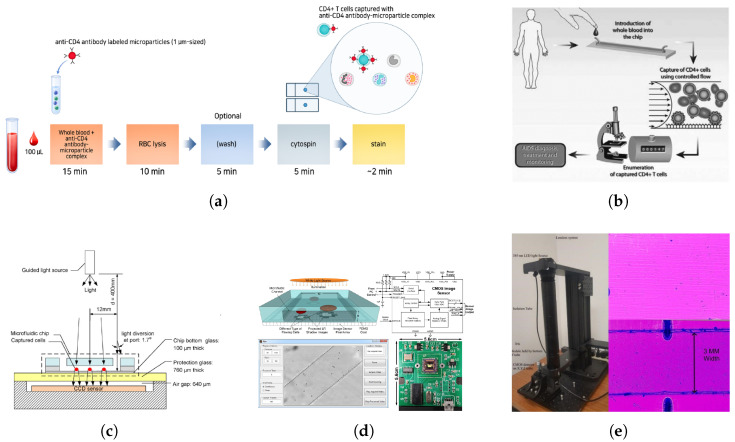
(**a**) ImmunoSpin system showing CD4 T cells tagged with microparticles for bright-field imaging, allowing morphology-based CD4 counting without fluorescence labeling [70]. (**b**) Label-free CD4 T cell isolation in a microfluidic device using cell affinity chromatography under controlled shear stress, enabling optical detection for POC applications [71]. (**c**) Lensless shadow imaging in a microfluidic device with anti-CD4 antibodies capturing target cells, producing grayscale shadows for rapid, label-free CD4 counting [73]. (**d**) Contact-imaging cytometer with ELM-SR machine learning for super-resolution processing, enhancing accuracy in real-time, high-throughput CD4 counting [74]. (**e**) Wide-field optical imaging system with automated CD4 counting software, using ImageJ-based analysis to boost throughput and sensitivity in POC diagnostics [75].

In Figure 5a, the ImmunoSpin method uses light microscopy to detect CD4+ T cells tagged with anti-CD4 antibody-conjugated microparticles, facilitating bright-field imaging without the need for fluorescence or microfluidic devices. By lysing red blood cells and concentrating leukocytes through cytocentrifugation, CD4+ cells become distinguishable under light microscopy, achieving accuracy comparable to clinical flow cytometry in resource-limited settings [70]. Additionally, in Figure 5b, Cheng et al. developed a microfluidic device for low-cost, label-free detection of CD4+ T cells using cell affinity chromatography operated under controlled shear stress [71]. The device requires only 10 µL of unprocessed whole blood, which is injected directly into a channel functionalized with anti-CD4 antibodies. Under optimized shear stress conditions (1–3 dyn/cm²), CD4+ T cells selectively adhere to the surface due to their higher expression of CD4 receptors compared to monocytes and other cells [71]. To differentiate CD4+ T cells from monocytes, the device leverages the physical and biological properties of these cells. Monocytes, which also express CD4, exhibit significantly lower adhesion efficiency at shear stresses above 0.7 dyn/cm² due to their larger size and lower CD4 receptor density. This shear stress window ensures that more than 95% of the captured cells are CD4+ T lymphocytes, with minimal contamination from other cell types. Captured cells are counted directly under a light microscope, using their specific adhesion to the antibody-functionalized surface as an identity marker. Unlike traditional flow cytometry, this method does not require fluorescent labeling, simplifying the process and reducing costs [71]. Comparative tests demonstrated strong agreement with conventional flow cytometry (R² = 0.93), confirming the device’s accuracy and potential as a practical tool for POC applications in resource-limited settings [43,71]. Imaging-based optical sensing has been applied to study red blood cells in Tanzania [72]. Researchers measured the morphological and biochemical properties of RBCs by transforming existing microscopes into quantitative phase microscopes.

In Figure 5c, Moon et al. introduced a lensless imaging platform that captures grayscale shadows of CD4+ cells bound to an anti-CD4 antibody-coated microfluidic chip [73]. This CCD-based lensless technique captures images rapidly, enabling automatic cell counting in under 10 min with a capture efficiency of 70.2% and a detection specificity of 88.8% relative to flow cytometry. This integrated system significantly reduces complexity and cost, making it well suited for POC testing in low-resource settings [73].

Further innovations include a contact-imaging microfluidic cytometer by Huang et al., shown in Figure 5d, which combines contact imaging with machine-learning algorithms for enhanced resolution and accuracy [74]. Single-frame super-resolution processing enables high-throughput, real-time cell analysis in continuous flow. The system achieved a counting accuracy within an 8% error margin compared to flow cytometry, illustrating its potential as a rapid and effective diagnostic tool.

Lastly, in Figure 5e, Fennell et al. developed a wide-field optical imaging system paired with ImageJ software (Wayne Rasband, National Institutes of Health, https://github.com/imagej/ImageJ) for automated CD4 counting on a microfluidic chip. This system achieves a high capture efficiency (98.3%) and specificity (89.3%) for CD4+ cells and can process larger sample areas in a single view, enhancing throughput and reliability in POC settings. Future integration of AI for more precise cell type discrimination could further improve specificity, making it an ideal tool for rapid and cost-effective CD4 enumeration in remote locations [75,76].

### 3.3. Absorbance-Based Optical Sensing

Absorbance-based optical sensing is a technique used to determine CD4 T lymphocyte concentrations by measuring light absorption at specific wavelengths. This allows for the indirect quantification of cell concentration based on the amount of light passing through the sample versus the amount absorbed. This approach leverages the fact that certain biomolecules or markers unique to CD4 cells absorb light in predictable ways, simplifying detection without the need for complex equipment such as lasers or fluorescence detectors. This makes absorbance-based sensing particularly advantageous for point-of-care (POC) applications, especially in resource-limited settings where access to advanced instrumentation is limited. Being less equipment-intensive, this method is ideal for decentralized healthcare.

**Figure 6:**
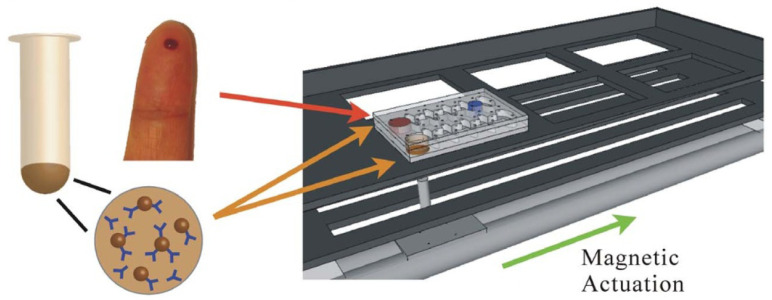
A droplet from a finger prick can be collected and loaded onto a micro-a-fluidic chip along with antibody-functionalized magnetic beads. The micro-a-fluidic chip is placed on a permanent magnet fixed on a motorized stage. With the aid of a software program, the stage is used to control and complete the entire process of ELISA in an automated manner [77].

The micro-a-fluidic ELISA technique represents an innovative adaptation of this principle for rapid CD4 cell counting. This approach, shown in Figure 6, is designed for POC use and automates enzyme-linked immunosorbent assay (ELISA) processing within a microfluidic channel [77]. By immobilizing anti-CD4 antibodies on magnetic beads, this platform captures CD4+ T lymphocytes directly from whole blood, eliminating the complex fluidic operations typically seen in conventional ELISA setups. Instead of flowing the substrate, the micro-a-fluidic ELISA moves the magnetic beads through different reagent chambers, significantly reducing the risk of air bubbles and flow control issues. The colorimetric readout is captured by a smartphone, providing highly accurate counts within minutes, making this an efficient solution for ART monitoring in resource-constrained areas [77,78].

### 3.4. Colorimetric Optical Sensing

**Figure 7:**
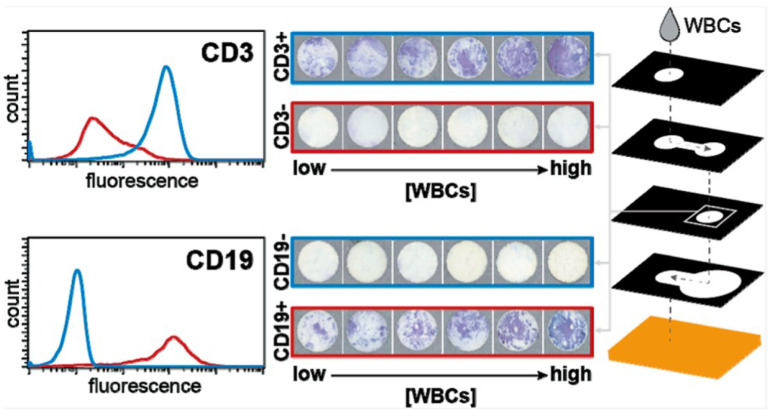
Device schematic showing each layer, its function, and the material used to fabricate that layer. The added sample contains a suspension of WBCs, which wicks vertically through the sample addition/cell labeling layer, interacting with the antibody conjugate that is stored there. The cells wick to the subsequent incubation layer, where they mix and bind to the antibody conjugate, given that the target cell type is present in the sample. The cells are retained by size on the readout layer, a PES membrane with a pore size of 0.8μm, and the remaining fluid wicks through the wash layer to the blot layer below. Representative scans from the calibration curve for CD3+ T and CD19+ B cells [79].

Colorimetric methods rely on visual color changes triggered by CD4 cell-specific chemical reactions. Colorimetric optical sensing for CD4 counting is a technique that uses color changes to detect and quantify CD4+ T lymphocytes. This method typically involves a chemical reaction that produces a visible color shift when CD4+ cells are present in a sample. The color intensity correlates with the concentration of CD4 cells, allowing for easy visual or instrumental measurement. These systems are simple to use but face limitations in detecting low CD4 concentrations due to limited sensitivity. One current constraint of POC white blood cell (WBC) counting devices is their “inherent limitation in supporting the detection of WBCs—the pore sizes of materials used to fabricate these devices do not permit passive WBC transport via wicking [79]”. Murray and Mace identified a new paper-based microfluidic device (Figure 7) capable of transporting WBCs both laterally and vertically. By using CEM-CD4+ T cells, a kind of leukocyte cell line, Murray and his colleagues successfully detected and enumerated the CD4+ T lymphocyte subset, which helps identify people with severe HIV disease/AIDS. Although their equipment functions as planned with cultured cells, further research and testing are required with whole blood, which is the optimal sample matrix for WBC enumeration. The ultimate device format aims to process fingerstick blood samples and provide semi-quantitative or quantitative WBC counts to the end user [79]. The colorimetric sensing has been successfully tested in seven countries, including Malawi, Tanzania, South Africa, Thailand, Uganda, Vietnam, and Zambia, with a sensitivity of 92.7% compared to the gold standard [80].

In Table 3, a comparison of optical sensing techniques for CD4 T cell enumeration is presented. These methods encompass fluorescence-based, imaging-based, absorbance-based, and colorimetric approaches, each offering unique strengths and limitations. Fluorescence-based techniques excel in sensitivity and specificity, making them suitable for high-precision applications. Imaging-based methods leverage advanced optics and machine learning algorithms for accurate real-time analysis, while absorbance- and colorimetric-based approaches prioritize simplicity and affordability, particularly for resource-limited settings. Collectively, these techniques address diverse diagnostic needs in sensitivity, specificity, and operational complexity, providing adaptable solutions for both clinical and POC applications.

## 4. Conclusions

Accurately counting CD4+ T lymphocytes is crucial for managing HIV/AIDS, especially in resource-limited settings where access to advanced diagnostic facilities is scarce. This review highlights the rapid advancements in microfluidic and point-of-care (POC) technologies designed to address the limitations of traditional flow cytometry. Integrating diverse sensing approaches—including electrical impedance and optical methods (fluorescence, imaging, absorbance, and colorimetric)—are revolutionizing CD4 enumeration. Aligned with the ASSURED criteria (Affordable, Sensitive, Specific, User-friendly, Rapid, Equipment-free, and Deliverable), these technologies are particularly suited for deployment in decentralized healthcare settings.

Each sensing modality presents unique strengths and challenges. Optical methods, such as fluorescence and imaging-based sensing, excel in sensitivity and specificity but often require precise alignment and entail higher costs. Electrical impedance techniques offer label-free, rapid analysis, but the complexity of biological samples can impact their performance. Despite the diversity of approaches, fluorescence-based optical sensing remains the most widely adopted technique. Commercialized systems, including BD FACSPresto, PointCare NOW™, Helios, and PIMA, have demonstrated their effectiveness and practicality for CD4 monitoring in clinical and point-of-care applications.

Future research should focus on improving these systems’ robustness, sensitivity, and affordability, emphasizing integrating advanced algorithms and smartphone-based interfaces to enhance usability in remote settings. As microfluidic POC devices continue to evolve, they have the potential to democratize access to CD4 monitoring, significantly improving patient outcomes and advancing global HIV/AIDS management. Additionally, clinical trials and further investigations into existing techniques, such as the PIMA evaluations in South Africa, should be prioritized to validate their effectiveness and expand their applicability. 

## Figures and Tables

**Table 1:** Comparison of electrical impedance sensing methods.

Method	Strengths	Limitations
Coulter Principle	High-speed, accurate size/count measurement	Inability to identify cell subtypes, clogging issues
Label-Free Impedance	Cost-effective, portable, easy fabrication	Limited to basic electrical properties
Electrical Impedance Spectroscopy (EIS)	High sensitivity, detailed cell analysis	Requires low-conductivity environments

**Table 2:** Comparison of commercial fluorescence-based optical sensing CD4 analyzers.

Aspect	BD FACSPresto	PointCare NOW™	Helios CD4 Analyzer	PIMA CD4 Analyzer
Sample Requirement	50 µL whole blood (capillary or venous)	50 µL whole blood (capillary or venous)	20–50 µL whole blood	10 µL whole blood (capillary or venous)
Detection Limit	50 cells/µL	100 cells/µL	100 cells/µL	50 cells/µL
Time to Results	5 min	20 min	10–15 min	20 min
Output Parameters	Absolute CD4 count, CD4 percentage, Hemoglobin concentration	Absolute CD4 count, White blood cell (WBC) count, Hemoglobin levels (via CBC)	Absolute CD4 count, CD4/CD8 ratio (optional)	Absolute CD4 count, CD4 percentage

**Table 3:** Comparison of Optical Sensing Techniques.

Technique	Advantages	Limitations
Fluorescence-Based	High specificity; robust for low cell counts; widely validated in POC settings	Requires precise optical alignment; expensive reagents
Imaging-Based	High resolution; adaptable to machine learning; minimal sample preparation	Sensitive to environmental light; dependent on professional medical staff
Absorbance-Based	Equipment-light; cost-effective for resource-limited settings	Limited specificity for CD4 counts; prone to signal interference
Colorimetric	Simplified detection; visual or smartphone-based readouts	Low sensitivity for sparse CD4 populations

## Data Availability

Not applicable.
